# Influence of Upper-Body Exercise on the Fatigability of Human Respiratory Muscles

**DOI:** 10.1249/MSS.0000000000001251

**Published:** 2017-03-10

**Authors:** NICHOLAS B. TILLER, IAN G. CAMPBELL, LEE M. ROMER

**Affiliations:** ^1^Academy of Sport and Physical Activity, Sheffield Hallam University, Sheffield, UNITED KINGDOM; ^2^Division of Sport, Health and Exercise Sciences, Brunel University London, London, UNITED KINGDOM; and ^3^School of Life and Medical Sciences, University of Hertfordshire, Hertfordshire, UNITED KINGDOM

**Keywords:** ABDOMINALS, ARM CRANKING, ARM EXERCISE, DIAPHRAGM, NERVE STIMULATION

## Abstract

**Purpose:**

Diaphragm and abdominal muscles are susceptible to contractile fatigue in response to high-intensity, whole-body exercise. This study assessed whether the ventilatory and mechanical loads imposed by high-intensity, upper-body exercise would be sufficient to elicit respiratory muscle fatigue.

**Methods:**

Seven healthy men (mean ± SD; age = 24 ± 4 yr, peak O_2_ uptake [V˙O_2peak_] = 31.9 ± 5.3 mL·kg^−1^·min^−1^) performed asynchronous arm-crank exercise to exhaustion at work rates equivalent to 30% (heavy) and 60% (severe) of the difference between gas exchange threshold and V˙O_2peak_. Contractile fatigue of the diaphragm and abdominal muscles was assessed by measuring pre- to postexercise changes in potentiated transdiaphragmatic and gastric twitch pressures (P_di,tw_ and P_ga,tw_) evoked by supramaximal magnetic stimulation of the cervical and thoracic nerves, respectively.

**Results:**

Exercise time was 24.5 ± 5.8 min for heavy exercise and 9.8 ± 1.8 min for severe exercise. Ventilation over the final minute of heavy exercise was 73 ± 20 L·min^−1^ (39% ± 11% maximum voluntary ventilation) and 99 ± 19 L·min^−1^ (53% ± 11% maximum voluntary ventilation) for severe exercise. Mean P_di,tw_ did not differ pre- to postexercise at either intensity (*P* > 0.05). Immediately (5–15 min) after severe exercise, mean P_ga,tw_ was significantly lower than pre-exercise values (41 ± 13 vs 53 ± 15 cm H_2_O, *P* < 0.05), with the difference no longer significant after 25–35 min. Abdominal muscle fatigue (defined as ≥15% reduction in P_ga,tw_) occurred in 1/7 subjects after heavy exercise and 5/7 subjects after severe exercise.

**Conclusions:**

High-intensity, upper-body exercise elicits significant abdominal, but not diaphragm, muscle fatigue in healthy men. The increased magnitude and prevalence of fatigue during severe-intensity exercise is likely due to additional (nonrespiratory) loading of the thorax.

The diaphragm and abdominal muscles of healthy human beings exhibit contractile fatigue after whole-body exercise sustained to exhaustion at intensities greater than 80% maximum O_2_ uptake (V˙O_2max_). Such exercise-induced respiratory muscle fatigue has been documented after cycle ergometry and treadmill running, whereby transdiaphragmatic (P_di_) and gastric (P_ga_) pressures evoked by supramaximal magnetic stimulation of the phrenic and thoracic nerves, respectively, were reduced by 15%–30% relative to preexercise values and took approximately 2 h to recover (20,35). The magnitude and the prevalence of diaphragmatic fatigue were significantly correlated with the ventilatory demands of exercise (20). Moreover, fatigue of the diaphragm was prevented when exercise-induced diaphragmatic work was reduced using a proportional assist ventilator (5). Together, these findings suggest that diaphragmatic fatigue is, in part, due to the high work of breathing that must be sustained throughout intense, whole-body exercise.

By contrast, contractile fatigue of the diaphragm did not occur when rested subjects mimicked the magnitude and duration of diaphragmatic work incurred during whole-body exercise; fatigue was only observed when diaphragmatic work was voluntarily increased twofold greater than that required during maximal exercise (4). Furthermore, there are no correlations between the magnitude of abdominal muscle fatigue and the ventilatory requirements of exercise. (35,40). Collectively, these findings suggest that mechanisms other than ventilatory load must contribute to the development of exercise-induced respiratory muscle fatigue. One possible mechanism is competition between respiratory and locomotor muscles for the limited available cardiac output during intense, whole-body exercise (14). Less blood flow to the respiratory muscles would promote inadequate O_2_ transport, thereby contributing to the development of fatigue.

The cardioventilatory demands of intense, upper-body exercise are considerably less than for whole-body exercise (34). These relatively low demands for ventilation and blood flow would be expected to reduce the likelihood of respiratory muscle fatigue during upper-body exercise. However, upper-body tasks place additional mechanical loads on the thoracic complex. For example, the respiratory muscles function to ventilate the lungs while simultaneously stiffening the spine (16,17) and maintaining torso stabilization and arm position (9). Specifically, the diaphragm aids trunk stability before rapid arm movements (15), and the abdominals contract to dynamically flex and rotate the torso (10). Given the combined ventilatory, postural, and locomotor functions of the diaphragm and abdominals, these muscles likely undergo substantial contractile work during upper-body exercise, possibly predisposing to contractile fatigue. Although previous studies have demonstrated significant reductions in volitional measures of respiratory muscle function after activities involving the upper body (e.g., rowing [41] and swimming [24]), such measures are highly dependent on subject motivation and cannot be used to infer a reduction in contractile function of the respiratory muscles.

Accordingly, the aim of the present study was to assess the fatigability of respiratory muscles, using nonvolitional (effort independent) motor nerve stimulation techniques, after sustained, intense, upper-body exercise in healthy subjects. It was hypothesized that 1) the additional mechanical demands imposed by upper-body exercise would induce contractile fatigue of the diaphragm and abdominal muscles and 2) the magnitude and prevalence of respiratory muscle fatigue would be dependent upon exercise intensity.

## METHODS

### Subjects

After providing written informed consent, seven healthy, nonsmoking men between the ages of 18 and 35 yr volunteered to participate in the study (mean ± SD; age = 24 ± 4 yr, stature = 1.77 ± 0.06 m, body mass = 75.5 ± 6.3 kg). The subjects were physically active but not engaged in specific upper-body exercise training. Experimental procedures were approved by the institutional research ethics committee. Subjects were asked to abstain from exercise for 48 h, alcohol and caffeine for 12 h, and food for 3 h before each visit.

### Experimental Overview

Each subject visited the laboratory on four separate occasions within a 2-wk period and with at least 48 h between visits. At the first visit, subjects underwent pulmonary function testing and were thoroughly familiarized with the neuromuscular function and arm-crank exercise protocols. At the second visit, subjects completed maximal incremental, asynchronous arm-crank exercise for the determination of gas exchange threshold and peak O_2_ uptake (V˙O_2peak_). The subsequent two visits were the experimental trials, which were performed in a random order and at the same time of day. The experimental trials comprised constant-load arm-crank exercise to exhaustion at heavy and severe intensities with the assessment of cardiorespiratory, metabolic, perceptual, and respiratory neuromechanical responses. Contractile fatigue of the diaphragm and abdominal muscles was assessed in both experimental trials by measuring the pre- to postexercise change in transdiaphragmatic twitch (P_di,tw_) and gastric twitch pressures (P_ga,tw_) in response to magnetic stimulation of the phrenic and thoracic nerves, respectively.

### Pulmonary Function, Visit 1

Pulmonary volumes, capacities, flows, resistance, and diffusion were assessed using a fully integrated system (Masterscreen; CareFusion, Hampshire, UK). Maximum inspiratory pressure at residual volume (RV) and maximum expiratory pressure at total lung capacity (TLC) were assessed using a handheld device (MicroRPM, CareFusion).

### Maximal Incremental Exercise, Visit 2

Subjects completed a maximal incremental exercise test on an electromagnetically braked arm-crank ergometer set in the hyperbolic mode (Angio; Lode, Groningen, The Netherlands). The ergometer was wall mounted and positioned so that the scapula-humeral joint and the distal end of the crank pedal were horizontally aligned. Subjects were instructed to sit upright, maintain form at all times, and keep their feet flat to the floor to minimize bracing. After 3 min of rest, subjects exercised for 3 min at 20 W after which the work rate was increased in a ramp fashion by 15 W·min^−1^. Cadence was standardized at 75 rpm to reflect the spontaneously chosen cadence of subjects performing maximal arm cranking (32). The test was terminated when cadence dropped below 65 rpm for more than 3 s despite verbal encouragement. Cardiorespiratory variables were assessed continuously, and peak values were reported as the highest 30-s average. The gas exchange threshold was identified independently by two investigators using multiple parallel methods (6).

### Constant-Load Exercise, Visits 3 and 4

After 3 min of rest and 3 min of light arm-crank exercise at 20 W, subjects abruptly transitioned to a work rate equivalent to 30% or 60% of the difference between gas exchange threshold and V˙O_2peak_ (i.e., the work rate at gas exchange threshold plus 30% or 60% of the difference between the work rate at gas exchange threshold and the work rate at V˙O_2peak_). The absolute work rates were reduced by 10 W (two-thirds of the initial ramp rate) to accommodate the mean lag time in V˙O_2_ that occurs during ramp exercise (42). The final work rates (Δ30% and Δ60%) were expected to result in physiological responses that are consistent with heavy- and severe-intensity exercise, respectively (23). Cadence was fixed at 75 rpm, and the test was terminated if cadence dropped below 65 rpm for more than 3 s or if exercise duration exceeded 30 min.

#### Cardiorespiratory, metabolic, and perceptual responses

Continuous measures of heart rate were made by telemetry (Vantage NV; Polar Electro, Kempele, Finland), arterial oxygen saturation (SpO_2_) by forehead pulse oximetry (OxiMax N-560; Nellcor, Tyco Healthcare, Pleasanton, CA), and pulmonary ventilation and gas exchange via online gas analysis (Oxycon Pro, CareFusion). Lactate concentration in whole-blood ([BLa]) was measured at resting baseline and immediately postexercise via a 10-μL earlobe capillary sample (Biosen C-Line; EKF Diagnostic GmbH, Barleben, Germany). Intensity of breathing discomfort (dyspnea) and intensity of limb discomfort were rated using Borg's modified CR10 scale (7). Perceptual responses to exercise were assessed on alternate minutes and at the point of exhaustion.

#### Respiratory neuromuscular responses

##### EMG and pressures

Neuromuscular activation of the crural diaphragm (EMG_di_) was assessed using a bespoke multipair esophageal electrode catheter (Gaeltec Devices Ltd., Dunvegan, Isle of Sky, UK). The catheter comprised a 100-cm silicon shaft (2.7 mm diameter) with seven platinum electrodes spaced 1 cm apart. Esophageal pressure (P_es_) and gastric pressure (P_ga_) were measured using two independent pressure transducers that were integrated with the esophageal catheter and positioned proximally and distally to the electrodes. The transducers were calibrated across the physiological range by placing the catheter within a sealed, air-filled tube to which positive and negative pressures were applied using a glass syringe (33). The calibration tube was connected to an electromanometer (C9553; Comark, Norwich, Norfolk, UK), and the voltage outputs of each pressure transducer were calibrated against the reference pressures. The catheter was passed pernasally into the stomach until the diaphragm produced a positive pressure deflection on inspiration and repositioned based on the strength of EMG_di_ recorded simultaneously from different pairs of electrodes. Neuromuscular activation of the rectus abdominis (EMG_ra_) was assessed using a pair of wireless surface electrodes (Trigno Wireless EMG; Delsys Inc., Natick, MA), positioned on the main belly of the muscle, 2 cm superior and 2–4 cm lateral to the umbilicus on the right-hand side of the torso, and placed in the same orientation as the muscle fibers (35). Raw EMG data were converted to root mean square using a time constant of 100 ms and a moving window. All EMG data were expressed as a percentage of maximum EMG activity recorded during any maximal inspiratory or expiratory maneuver performed at rest or during exercise for a given experimental visit.

##### Respiratory mechanics and operating lung volumes

An analog airflow signal from the online gas analysis system was input into the data acquisition system (see Data Processing section) and aligned to the pressure signals based on the sampling delay for flow. Transdiaphragmatic pressure (P_di_) was obtained by online subtraction of P_es_ from P_ga_. Tidal pressure swings (ΔP_es_, ΔP_ga_, and ΔP_di_) were calculated as the changes in pressure from points of zero flow. Maximum pressures (P_es,max_, P_ga,max_, and P_di,max_) were obtained from maximal static inspiratory and expiratory maneuvers performed immediately before exercise. The work of breathing (*W*_b_; work done per minute on the lungs) was calculated as the integral of the P_es_–volume loop multiplied by respiratory frequency. Our assessment of *W*_b_ does not include work performed on the chest wall (e.g., rib cage distortion) and might, therefore, underestimate the mechanical work of breathing during exercise. Operating lung volumes were assessed using an inspiratory capacity (IC) maneuver performed in duplicate at resting baseline and during the final 30 s of alternate minutes of exercise starting at the first minute. Verbal encouragement was given to ensure a maximal inspiratory effort, and the maneuver was considered acceptable when ΔP_es_ matched that achieved at baseline. End-expiratory lung volume (EELV) was calculated by subtracting IC from TLC. End-inspiratory lung volume (EILV) was calculated as the sum of tidal volume and EELV. Both EELV and EILV were expressed relative to TLC. To quantify the degree of diaphragm and abdominal muscle function during exercise that was due to locomotor (nonrespiratory) loading, we compared intrathoracic pressure swings and respiratory muscle EMG responses during five respiratory cycles at peak exercise to those recorded during five respiratory cycles immediately after exercise cessation.

#### Neuromuscular stimulation

A monophasic magnetic stimulator (Magstim 200; The Magstim Company Ltd., Whitland, Wales) was used to deliver magnetic stimuli to the spinal foramina. A circular 90-mm coil was positioned at the cervical or thoracic spinal nerve roots to discriminate between the diaphragm and the abdominal muscles, respectively (22,29). The stimulator was discharged at 100% when subjects were rested at functional residual capacity with the glottis closed. The catheter-mounted pressure transducer used in the current study has been shown to exhibit baseline drift after several hours of use (33). To minimize the extent of drift, the catheter was soaked in water for 1 h before use and the amplifier gain settings were lowered. Furthermore, the pressure traces were checked immediately before stimulation and discarded if unstable. Cervical stimulation was favored over anterolateral stimulation of the phrenic nerves as it allows costimulation of the diaphragm and ribcage muscles, thereby allowing contractile fatigue of these muscles to be independently assessed (30). When stimulating the inspiratory muscles, subjects sat upright in a chair with their neck flexed and the coil positioned between the midline of the fifth (C5) and seventh (C7) cervical vertebrae. For the abdominal muscles, subjects sat facing an inclined bench (~70° from horizontal) with their chest supported, abdomen relaxed, and the coil positioned between the 8th (T8) and the 11th (T11) thoracic vertebrae. The coil position that evoked the highest P_di,tw_ or P_ga,tw_ upon stimulation was marked on the skin and used for all subsequent stimulations. To determine whether the respiratory muscles were maximally activated after the delivery of magnetic stimuli, three single twitches were applied to the cervical and thoracic regions at incremental percentages of maximum stimulator output (50%, 60%, 70%, 80%, 85%, 90%, 95%, and 100%). Each twitch was separated by 30 s to avoid potentiation. A plateau in mean P_di,tw_ and P_ga,tw_ was assumed to be indicative of maximal activation. Reliability of within-day measurements of respiratory muscle function was assessed by repeating baseline potentiated twitches after 30 min of quiet breathing. The order of diaphragm and abdominal muscle assessment was randomized, but consistent between trials for each subject.

Fatigue was quantified by measuring changes in neuromuscular function from preexercise baseline to postexercise (5–15 and 25–35 min). The potentiated twitch is the most sensitive and valid measure of fatigue when the degree of fatigue is small or when levels of postactivation potentiation are unequal. Therefore, P_di,tw_ and P_ga,tw_ were assessed in response to stimulation of the cervical or thoracic nerves immediately after a maximal inspiratory or expiratory pressure maneuver, respectively. The maximal inspiratory and expiratory maneuvers were initiated from RV and TLC, respectively, and were maintained for 5 s against a semi-occluded airway. The procedure was repeated five times, and the mean of the final three twitches was used for analysis. The primary outcome measure was the amplitude (baseline to peak) of the pressure response. Additional fatigue measures included contraction time and one-half relaxation time. Membrane excitability was determined by measuring the peak-to-peak amplitude and duration of magnetically evoked M-waves. Fatigue was considered to be present if there was a ≥15% reduction in P_di,tw_ or P_ga,tw_ relative to preexercise baseline values at any time after exercise (13). This conservative definition of fatigue is based on a change that is approximately two- to threefold greater than the typical variation in rested P_di,tw_ and P_ga,tw_ (see Results section).

### Data Processing

Cardiorespiratory data during constant-load exercise were averaged over alternate 30-s intervals when IC maneuvers were not being performed. Pressure signals were passed through an amplifier (1902; Cambridge Electronic Design, Cambridge, UK) and digitized along with airflow at a sampling rate of 150 Hz using an analog-to-digital converter (micro 1401 mkII, Cambridge Electronic Design). EMG signals were sampled at 4 kHz, high-pass filtered at 100 Hz, and notch filtered at 50 Hz to suppress power line and harmonic interference. Data were displayed as waveforms using data acquisition software (Spike 2 version 7.0, Cambridge Electronic Design). ECG artifacts were removed from the EMG waveforms using a script procedure similar to that described previously (3).

### Statistics

Statistical analysis was performed using SPSS 16.0 for Windows (IBM, Chicago, IL). Cardiorespiratory responses during heavy and severe exercise were assessed for differences using paired-samples *t*-test. Respiratory neuromuscular responses to constant-load exercise were assessed using two-way (intensity–time) repeated-measures ANOVA. Supramaximality of twitch responses and differences in respiratory neuromuscular function across time (preexercise, 10 and 30 min postexercise) were assessed using one-way repeated-measures ANOVA with Fisher's LSD for *post hoc* comparisons. Pearson's correlation coefficient (*r*) was computed to assess the relationship between pre- to postexercise percent changes in respiratory muscle function and selected parameters. Reliability of evoked pressures (P_di,tw_ and P_ga,tw_) was assessed using coefficient of variation (CV) and intraclass correlation coefficient (ICC). A two-tailed *α* level of 0.05 was used as the cutoff for statistical significance. Results are presented as means ± SD.

## RESULTS

### Pulmonary Function and Incremental Exercise

All subjects exhibited normal pulmonary function (VC [%pred], 5.76 ± 0.63 L [111% ± 10%]; FEV_1_, 4.99 ± 0.61 L [113% ± 11%]; FEV_1_/VC, 86.3% ± 2.7% [104% ± 3%]; TLC, 7.55 ± 0.77 L [106% ± 8%]; MVV_12_, 186 ± 14 L·min^−1^ [108% ± 9%]; D_L,CO_, 13.1 ± 1.6 mmol·min^−1^·kPa^−1^ [109% ± 12%]; P_Imax_, 132 ± 39 cm H_2_O [113% ± 37%]; P_Emax_, 128 ± 20 cm H_2_O [81% ± 14%]). Peak values for work rate and O_2_ uptake during maximal incremental exercise were 126 ± 25 W and 2.06 ± 0.41 L·min^−1^ (27.3 ± 3.3 mL·kg^−1^·min^−1^), respectively. Work rate and V˙O_2_ at gas exchange threshold were 60 ± 17 W and 1.17 ± 0.22 L·min^−1^ (57% ± 5% V˙O_2peak_). The work rates predicted to elicit 30% and 60% of the difference between gas exchange threshold and V˙O_2peak_ (Δ30% and Δ60%) were 69 ± 18 and 89 ± 20 W.

### Constant-Load Exercise

#### Cardiorespiratory, metabolic, and perceptual responses

**FIGURE 1:**
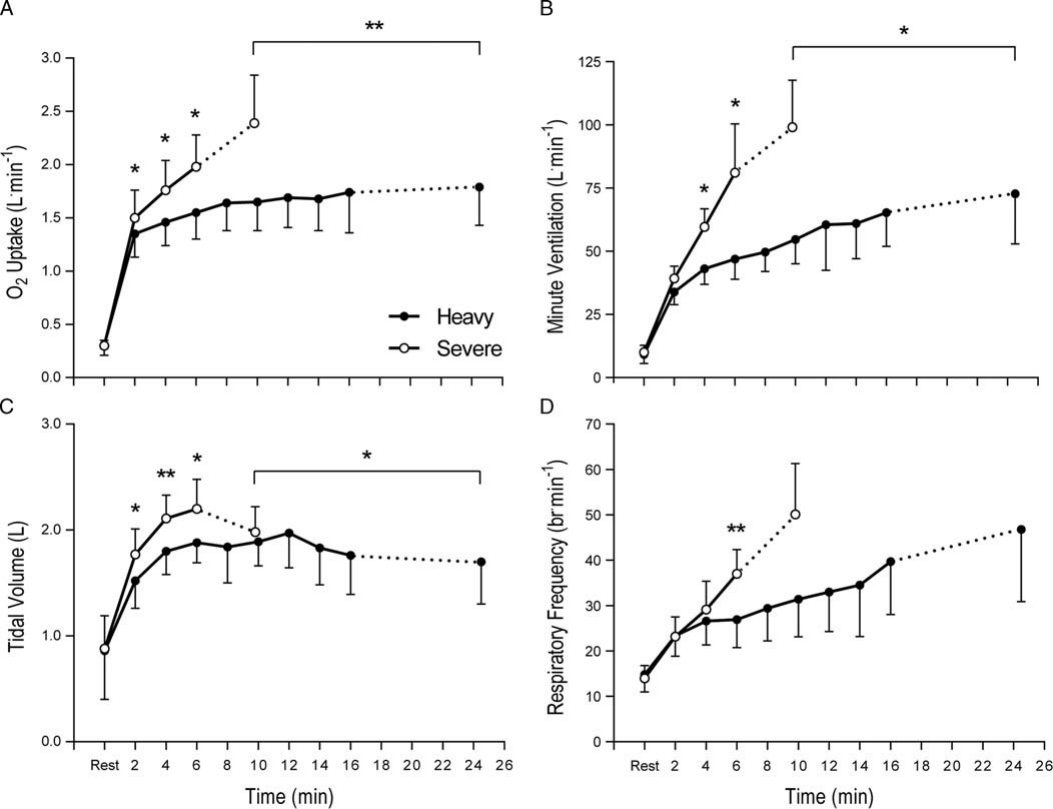
O_2_ uptake (A), minute ventilation (B), tidal volume (C), and respiratory frequency (D) at rest (0 min) and during constant-load arm-crank exercise at heavy (Δ30%) and severe (Δ60%) intensities. Values approached a steady-state during heavy exercise but continued to rise toward maximum values during severe exercise, except tidal volume, which showed a characteristic drop at near maximal ventilation. Data are presented as mean ± SD for seven subjects. *P < 0.05, **P < 0.01.

End-exercise values for heavy (Δ30%) and severe (Δ60%) exercise are shown in Table 1. Exercise duration was significantly shorter for severe compared with heavy exercise (*P* < 0.001). Three subjects reached the 30-min limit imposed for heavy exercise. However, all three subjects were considered to be close to their limit of tolerance as final minute responses and temporal profiles were similar to the remaining subjects. Heavy exercise elicited 81% ± 5% of the V˙O_2peak_ attained during incremental exercise, whereas severe exercise elicited values that were slightly, but not significantly, higher (108% ± 5%). Compared with heavy exercise, severe exercise elicited higher peak values for V˙O_2_ (*P* < 0.001), V˙CO_2_ (*P* < 0.001), RER (*P* = 0.025), V˙_E_ (*P* = 0.022), *V*_T_ (*P* = 0.043), *V*_T_/*T*_I_ (*P* < 0.001), heart rate (*P* = 0.005), and [BLa] (*P* = 0.006). Temporal profiles for V˙O_2_, V˙_E_, *V*_T_, and *f*_R_ during heavy and severe exercise are shown in Figure 1. During heavy exercise, V˙O_2_ and V˙_E_ rose sharply after exercise onset and increased at a slower rate thereafter. During severe exercise, V˙O_2_ and V˙_E_ continued to rise toward maximum values. At both intensities, the initial sharp rise in V˙_E_ was accounted for by progressive increases in *f*_R_ and *V*_T_, whereas during the latter stages of exercise, V˙_E_ was achieved primarily by increases in *f*_R_ (i.e., tachypnea). Minute ventilation over the final minute of heavy and severe exercise was 39% ± 11% and 53% ± 11% of maximum voluntary ventilation (MVV), respectively. Perceptual ratings of dyspnea were also higher during severe compared with heavy exercise (*P* = 0.042), as were the ratings of limb discomfort (*P* = 0.031). At the cessation of both exercise trials, five of seven subjects reported higher ratings for limb discomfort than for dyspnea, with two subjects rating both perceptions equal. When asked their reasons for stopping, all seven subjects cited arm fatigue and/or peripheral discomfort.

**TABLE 1:**
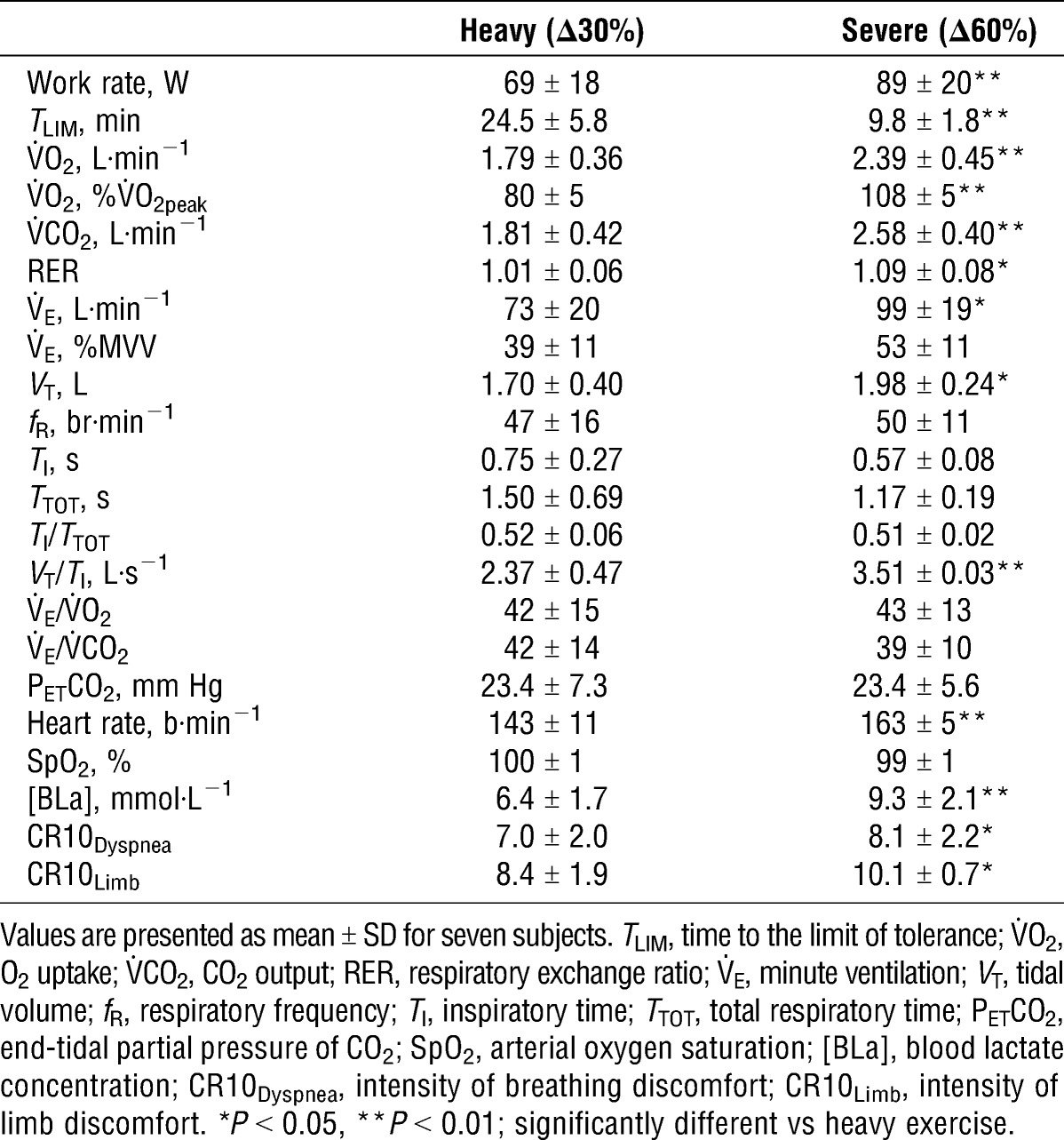
Cardiorespiratory, metabolic, and perceptual responses to maximal constant-load exercise.

#### Respiratory mechanics, EMG, and operating lung volumes

**FIGURE 2:**
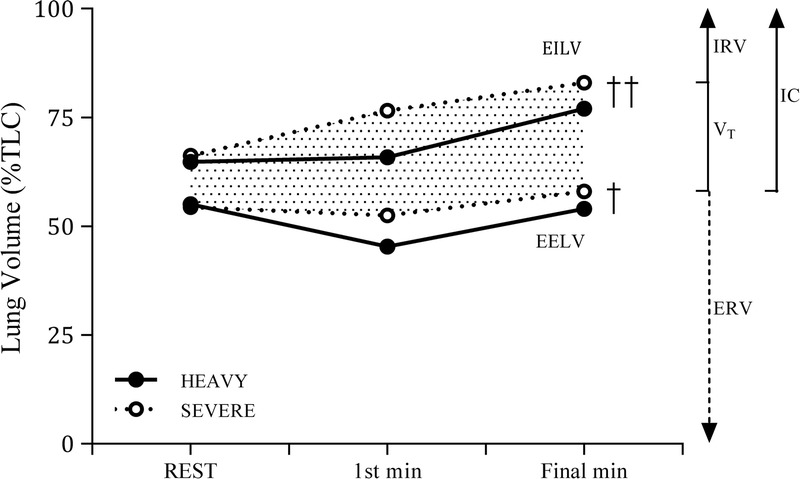
Operating lung volumes at resting baseline and during the first minute and final minute of heavy (Δ30%) and severe (Δ60%) constant-load arm-crank exercise. EELV and EILV were similar at rest in both conditions, but represented a higher percentage of TLC throughout severe exercise. During the final minute of severe exercise, EELV and EILV were elevated above rest, indicative of dynamic lung hyperinflation. IRV, inspiratory reserve volume; *V*_T_, tidal volume; ERV, expiratory reserve volume; IC, inspirtory capacity. Data are presented as mean for seven subjects. Error bars have been removed for clarity. †*P* < 0.05, ††*P* < 0.01; significant main effect for exercise intensity.

Group mean data for respiratory mechanics are shown in Table 2. Baseline pressure swings were not significantly different between the two trials. Mechanical responses over the final minute were significantly greater during severe exercise for ΔP_di,insp_ (*P* = 0.002), ΔP_ga,insp_ (*P* = 0.008), ΔP_es,insp_ (*P* = 0.001), ΔP_ga,exp_/P_ga,max_ (*P* = 0.005), ΔP_es,insp_/P_es,max_ (*P* = 0.003), and *W*_b_ (*P* = 0.027). EMG activity of the diaphragm and abdominal muscles tended to be higher during severe versus heavy exercise, although statistical significance was noted only for the abdominals (*P* = 0.20 and 0.003, respectively). Operating lung volumes at baseline and during the first and final minute of heavy and severe exercise are shown in Figure 2. Baseline values for EELV and EILV did not differ between heavy and severe exercise (EELV: 54% ± 6% vs 55% ± 3% TLC, *P* = 0.70; EILV: 66% ± 4% vs 65% ± 3% TLC, *P* = 0.42). During heavy exercise, EELV decreased below baseline then returned toward baseline as exercise progressed. By contrast, EELV at the first minute of severe exercise was similar to baseline and elevated above baseline by the final minute (i.e., dynamic hyperinflation). Both EELV and EILV were higher during severe compared with heavy exercise at the first minute (EELV: 52% ± 5% vs 45% ± 5% TLC; EILV: 77% ± 5% vs 66% ± 8% TLC) and at the final minute (EELV: 58% ± 3% vs 54% ± 7% TLC; EILV: 83% ± 7% vs 77% ± 6% TLC), with significant main effects for exercise intensity (EELV: *P* = 0.034; EILV: *P* = 0.009).

**TABLE 2:**
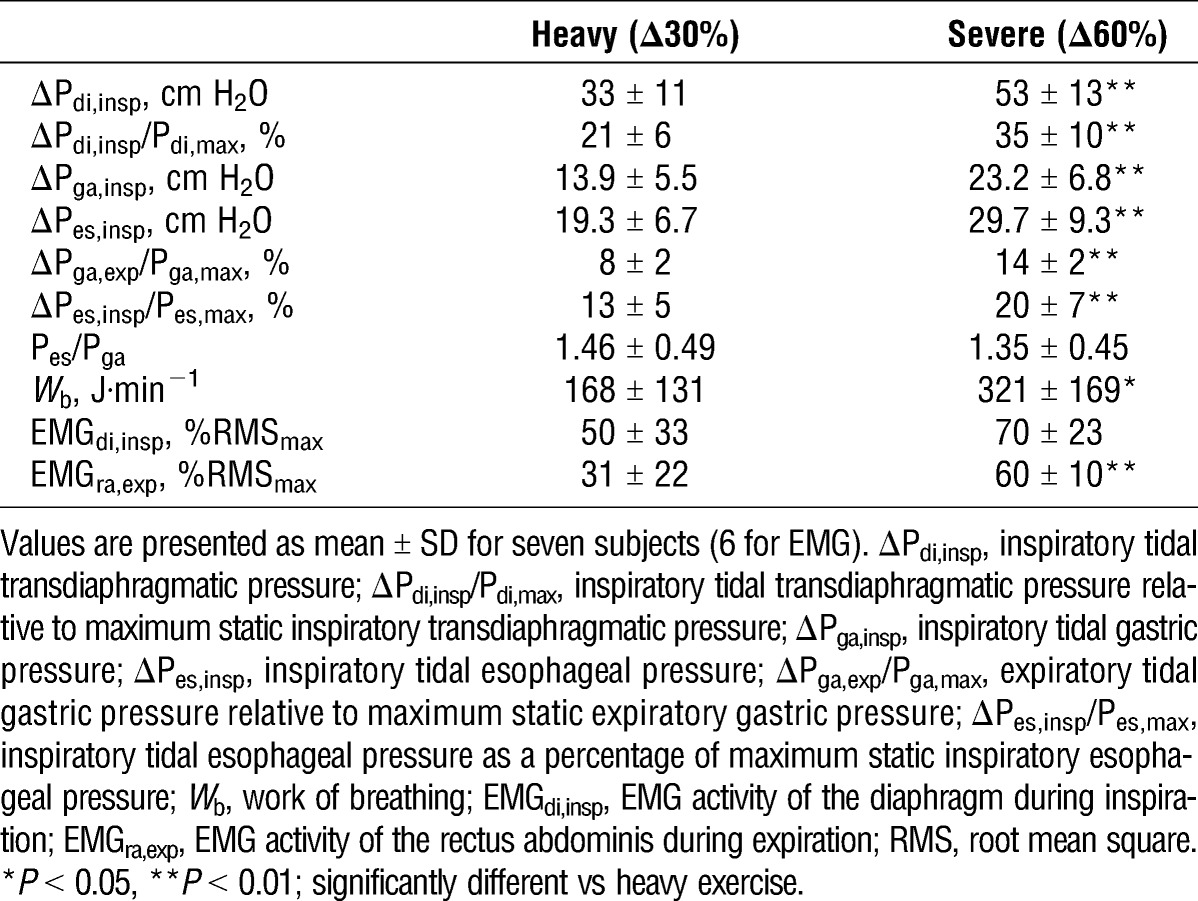
Peak respiratory neuromechanical responses to maximal constant-load exercise.

### Neuromuscular Function

#### Supramaximal stimulation and reliability

A near plateau in P_di,tw_ and P_ga,tw_ with increasing stimulator intensities was observed at baseline, with no significant differences in P_di,tw_ (*P* = 0.14) or P_ga,tw_ (*P* = 1.0) when the intensity was increased from 95% to 100%. There were no systematic differences in within-day, between-occasion measurements of respiratory muscle function, and reliability coefficients were <7% (CV) and >0.94 (ICC). Specifically, P_di,tw_ and P_ga,tw_ measured before and after 30 min of quiet rest were 56 ± 14 versus 54 ± 11 cm H_2_O (*P* > 0.05, CV = 5.4%, ICC = 0.96) and 59 ± 17 versus 56 ± 14 cm H_2_O (*P* > 0.05, CV = 7.3%, ICC = 0.94), respectively. Mean CV in P_di,tw_ at baseline and at 5–15 and 25–35 min after heavy exercise was 6.9%, 7.0%, and 5.4% for heavy exercise (*P* = 0.715) and 7.2%, 9.5%, and 5.3% for severe exercise (*P* = 0.240), respectively. Corresponding values for P_ga,tw_ were 5.4%, 6.2%, and 5.6% for heavy exercise (*P* = 0.860) and 5.9%, 9.9%, and 8.5% for severe exercise (*P* = 0.067). There were no significant differences at any time-point at either exercise intensity.

#### Pre- to postexercise responses

Data for neuromuscular function before and after exercise are shown in Table 3. Preexercise baseline values were not different between the two trials (heavy vs severe). No differences in diaphragm muscle contractility (P_di,tw_) or inspiratory ribcage muscle function (P_es,tw_/P_ga,tw_) were noted across time in either trial. Abdominal muscle contractility (P_ga,tw_) was also not different across time for heavy exercise (*P* = 1.0). After severe exercise, however, P_ga,tw_ was significantly reduced below baseline (−22% ± 18%, *P* = 0.038) and only partially recovered by 30 min (−15% ± 15%, *P* = 0.066). Analysis of the individual responses after severe exercise showed that five of seven subjects exhibited a ≥15% reduction in P_ga,tw_ (30% ± 15%) and two of seven subjects exhibited a ≥15% reduction in P_di,tw_. After heavy exercise, one of seven subjects exhibited a ≥15% reduction in P_ga,tw_ with no subjects exhibiting a reduction ≥15% in P_di,tw_. Within-twitch parameters (contraction time and one-half relaxation time) and M-wave characteristics (amplitude and duration) were not significantly different across time at either intensity. The degree of abdominal muscle fatigue (percent change in P_ga,tw_) immediately after severe constant-load exercise for all subjects correlated significantly and positively with both exercise duration (*r* = 0.82, *P* = 0.024) and peak [BLa] (*r* = 0.94, *P* = 0.002), but there were no significant correlations between the degree of fatigue after severe exercise and peak values for V˙O_2_ (*r* = 0.48, *P* = 0.28), V˙_E_ (*r* = 0.46, *P* = 0.30), or ΔP_ga_ (%max) (*r* = 0.34, *P* = 0.45). Finally, there were no remarkable differences in any of the ventilatory or neuromechanical responses between subjects exhibiting fatigue and those not.

**TABLE 3:**
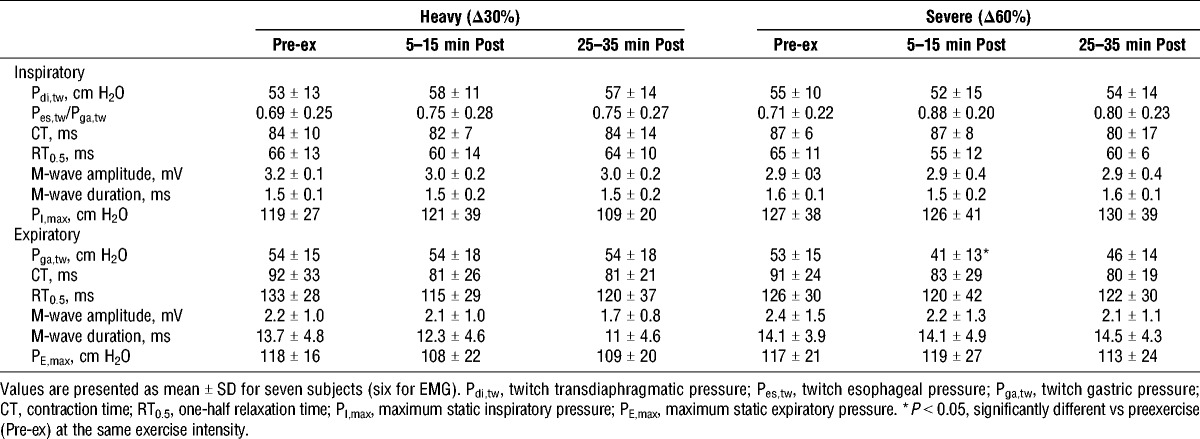
Neuromuscular function before and up to 30 min after constant-load exercise.

**FIGURE 3:**
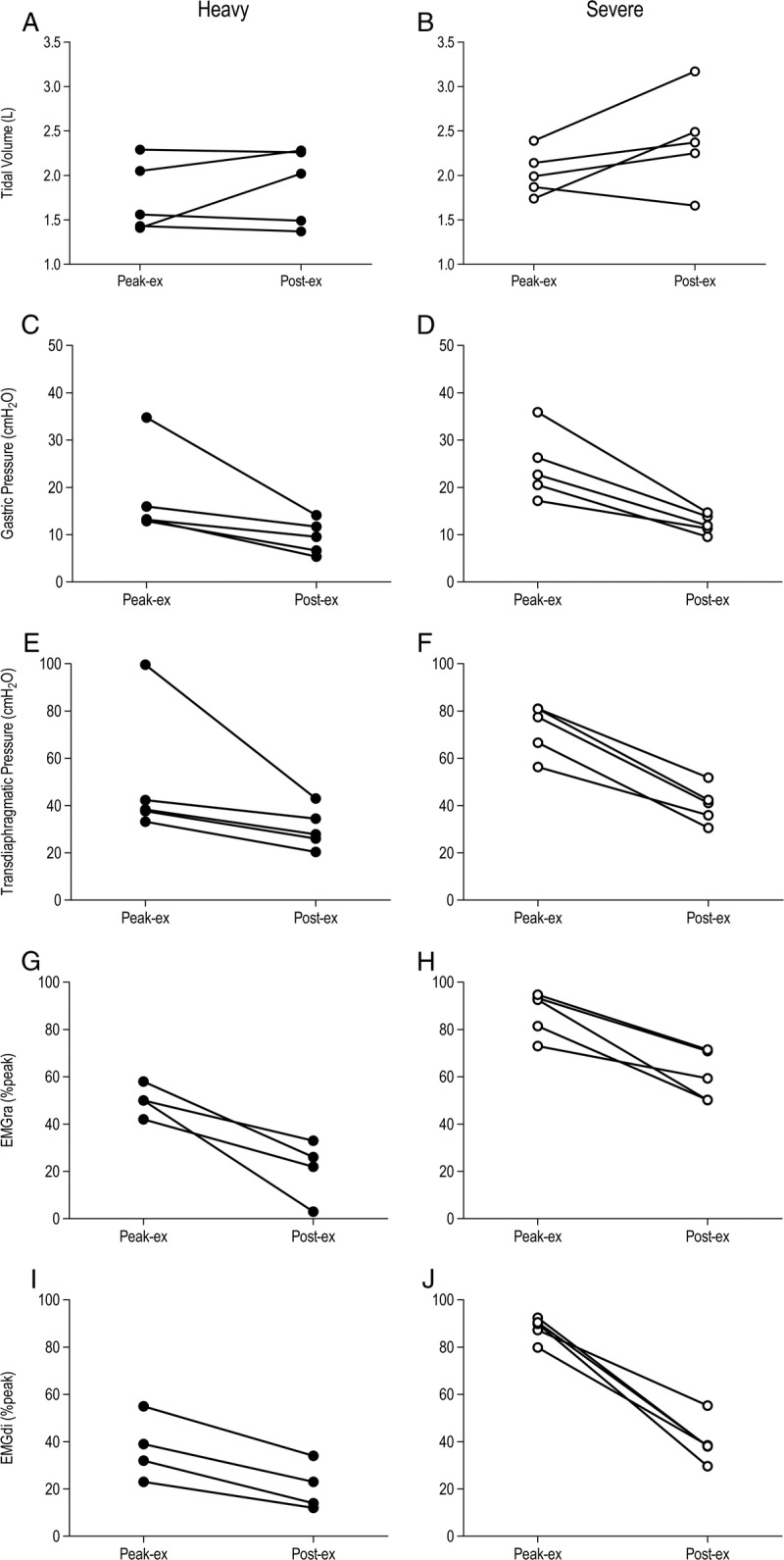
Respiratory responses during and immediately after exhaustive constant-load arm-crank exercise for the five subjects who exhibited abdominal muscle fatigue (≥15% reduction in P_ga,tw_). Data depict the mean of five respiratory cycles that preceded exercise cessation (peak-ex) compared with the first five respiratory cycles performed immediately after the abrupt cessation of exercise (post-ex). The left and right panels show data for heavy exercise and severe exercise, respectively; tidal volume (panels A and B), gastric pressure (panels C and D), transdiaphragmatic pressure (panels E and F), electromyographic activity of the rectus abdominis (panels G and H), and electromyographic activity of the diaphragm (panels I and J). One subject did not exhibit reliable EMG traces after heavy exercise, so data are for four subjects at this intensity. Note the abrupt increases in tidal volume despite substantial reductions in intrathoracic pressure swings and respiratory muscle EMG responses.

For the five subjects exhibiting a ≥15% reduction in abdominal muscle contractile function (P_ga,tw_), there were substantial differences in the peak-to-postexercise respiratory response (Fig. 3). Tidal volume increased immediately on cessation of both heavy exercise (1.75 to 1.88 L) and severe exercise (2.03 to 2.39 L). Gastric and transdiaphragmatic pressure swings dropped immediately after heavy exercise (ΔP_ga_, 18 to 10 cm H_2_O; ΔP_di_, 50 to 30 cm H_2_O) and severe exercise (ΔP_ga_, 25 to 12 cm H_2_O; ΔP_di_, 72 to 40 cm H_2_O). EMG activity of the rectus abdominis and diaphragm also fell substantially after heavy exercise (EMG_ra_, 50% to 21% peak; EMG_di_, 37% to 21% peak) and severe exercise (EMG_ra_, 87% to 60% peak; EMG_di_, 88% to 40% peak).

## DISCUSSION

### 

#### Main findings

This study is the first to use nonvolitional (effort independent) motor nerve stimulation techniques to assess the influence of upper-body exercise on the fatigability of respiratory muscles in normal, healthy subjects. The main finding was that severe (but not heavy) constant-load upper-body exercise impaired abdominal muscle contractility, as evidenced by a significant postexercise reduction in P_ga,tw_. By contrast, upper-body exercise did not influence the fatigability of the major muscles of inspiration, as demonstrated by nonsignificant changes in P_di,tw_ (diaphragm) and P_es,tw_/P_ga,tw_ (inspiratory rib cage muscles) after both heavy and severe exercise. The increased magnitude (and prevalence) of abdominal muscle fatigue associated with severe-intensity exercise might have been due to additional, nonrespiratory loading of the thoracic complex.

#### Exercise-induced respiratory muscle fatigue

Using objective measures of fatigue (i.e., evoked pressures in response to motor nerve stimulation), we found a significant reduction in the P_ga,tw_ response to magnetic stimulation of the thoracic nerves after severe, upper-body exercise. The time course of change in P_ga,tw_ was consistent with previous studies using whole-body exercise (35,40), with the greatest reduction observed within 5–15 min after exercise and partial recovery to baseline values by 25–35 min. The reduced P_ga,tw_ after severe exercise is indicative of low-frequency peripheral fatigue. The underlying mechanisms are thought to be reduced Ca^2+^ release from the sarcoplasmic reticulum, reduced Ca^2+^ sensitivity of the myofibrils, and/or damaged sarcomeres caused by overextension of the muscle fiber (21). Because P_ga,tw_ had partially returned to baseline by 25–35 min postexercise, the fatigue observed was likely due to reduced calcium release and/or sensitivity.

The magnitude of the postexercise reduction in P_ga,tw_ (22%) was similar to that noted by previous studies for intense, whole-body exercise (e.g., 33% [35], 26% [37], 25% [36], and 13% [40]), despite markedly lower levels of ventilation in the present study (99 vs 153, 138, 136, and 119 L·min^−1^). It is likely, therefore, that abdominal muscle fatigue is largely independent of ventilation and that, irrespective of the mechanical-ventilatory stress imposed by exercise, there is an upper limit for an acceptable reduction in abdominal muscle contractility beyond which exercise ventilation might be impaired. The interindividual fatigue response after severe exercise was variable, with five of seven subjects (~70%) exhibiting evidence of abdominal muscle fatigue (≥15% reduction in P_ga,tw_) and two of seven subjects (~30%) showing evidence of diaphragm fatigue (≥15% reduction in P_di,tw_). Abdominal muscle fatigue was present in one of seven subjects (~14%) after heavy exercise, whereas there was no evidence of diaphragm fatigue at this intensity. It is not entirely clear why fatigue was present in some subjects but not others. Subject characteristics were similar, and there were no remarkable fatigue-mediated differences in the ventilatory or neuromechanical responses to exercise. However, the significant correlation between the magnitude of fatigue and the exercise duration suggests that the prevalence of fatigue might have been related to the relative work capacity of the subject and, therefore, the absolute duration of exercise.

#### Methodological considerations

To be confident that nerve stimulation techniques provide a valid measure of fatigue, it is important to consider potential sources of error (see also Methods section). First, if pre- to postexercise changes in twitch pressure are to be attributed to contractile fatigue, then nerve stimulation must be supramaximal. In the present study, there was a trend toward a plateau in both P_di,tw_ and P_ga,tw_ with increasing stimulator output, with no significant differences between 95% and 100%. Although submaximal stimulation may underestimate the severity of fatigue (37), it seems unlikely that this would have influenced our finding of a difference in the magnitude and prevalence of fatigue for the diaphragm and abdominal muscles. Importantly, there were no significant changes in M-wave characteristics (amplitude and duration) for the stimulations delivered pre- to postexercise (see Table 3). This latter finding strongly suggests that the reductions in evoked pressure immediately after exercise were attributable to contractile fatigue rather than transmission failure or derecruitment of muscle fibers. In addition, all stimulations were performed at 100% of stimulator output, and the coil positions were marked before exercise to ensure the coil was repositioned in the same location for each stimulation. Therefore, although stimulation may not have been completely maximal, it likely remained constant throughout the study. A second potential source of error is the lung volume, and hence muscle length, at which stimulations are initiated (31). In the present study, it was not possible to use end-expiratory P_es_ for the verification of lung volume because of the baseline-drift inherent to the catheter-mounted pressure transducer (33). To enable lung volumes to return to baseline, postexercise stimulations were initiated at 5 min into recovery. Importantly, the repeatability of evoked pressures (P_di,tw_ and P_ga,tw_) was not significantly different before versus after exercise at either intensity. We are confident, therefore, that all stimulations were initiated at the same lung volume and that differences in lung volume did not account for the influence of exercise intensity on the magnitude and prevalence of respiratory muscle fatigue. Third, potentiated (rather than unpotentiated) twitches were used as these provide greater sensitivity when measuring a small degree of fatigue. Potentiated twitches are also more valid for detecting fatigue when there is a differing level of postactivation potentiation, as might be expected in the present study due to differences in exercise duration between trials and, thus, exercise-induced respiratory muscle activation. Despite lower than normal values for maximum expiratory pressure (P_E,max_), we are confident that the degree of potentiation was similar for the diaphragm and abdominal muscles. Indeed, previous studies have shown that twitch potentiation can be substantial after submaximal contractions (43) and that the degree of twitch potentiation is not significantly different between submaximal and maximal voluntary contractions (25). Fourth, the differences in fatigue noted in the present study were not merely a function of an inability to detect small within-day, between-trial changes in neuromuscular responses. There were no systematic differences in the measurements of respiratory muscle function when subjects were tested before and after 30 min of quiet breathing. Moreover, the within-day, between-occasion reliability of evoked pressures was excellent (CV range, 4.8%–7.3%) and similar to previously reported values: P_di,tw_, 5.6% (38); P_ga,tw_, 3.8% (35), 2.8% (37), and 7.0% (38). Thus, the methods used were likely sufficiently sensitive and reproducible to detect differences in exercise-induced respiratory muscle fatigue. A final consideration is the short-term recovery that likely occurs during the delay between end-exercise and postexercise evaluation of neuromuscular function. Although such a delay may underestimate the severity of fatigue during exercise (8), it is unlikely to explain the differences in the magnitude and prevalence of diaphragm and abdominal muscle fatigue noted in the current study.

#### Causes of fatigue

A time- and intensity-dependent increase in ventilation occurred during exercise (Fig. 1), requiring the progressive recruitment of inspiratory and expiratory muscles (Table 2). However, the overall ventilatory response was relatively low (<55% MVV). Furthermore, perceptual ratings of limb discomfort at end-exercise were higher than those reported for dyspnea, and all of the subjects cited limb fatigue and/or limb discomfort as their principal reasons for terminating severe exercise. These data are in general agreement with previous observations for maximal arm-crank exercise (28), suggesting that the exercise limitation was more closely associated with local (peripheral) than with central ventilatory factors. Because the majority of previous studies have only observed diaphragm fatigue at intensities exceeding 80% of whole-body V˙O_2max_ (see Introduction), it is perhaps unsurprising that arm-crank exercise did not induce contractile fatigue of the diaphragm. Our findings are in accordance with Taylor et al. (39) who found no evidence of diaphragm fatigue in athletes with spinal cord injury (C5–C7) performing exhaustive arm-crank exercise, during which ventilation peaked at <50 L·min^−1^. More recently, we observed a substantial (33%) reduction in P_di,tw_ in a Paralympic athlete with low-lesion spinal cord injury (T12) performing maximal arms-only rowing, during which ventilation peaked at >150 L·min^−1^ (27). Collectively, these data support the notion that high levels of pulmonary ventilation might be a prerequisite for exercise-induced diaphragm fatigue.

Because the diaphragm has both ventilatory and postural roles, it was expected that intense upper-body exercise would exacerbate diaphragm work, thereby leading to contractile fatigue, and yet this was not found. However, there is strong evidence to suggest that the diaphragm can only coordinate both postural and respiratory functions during transient, intermittent disturbances to trunk stability (e.g., brief arm movements) (19). When ventilation is mediated by humoral factors during sustained exercise, postural drive to the phrenic motoneurons is withdrawn, and pontomedullary respiratory input to the diaphragm is instead prioritized (18). During prolonged exercise, therefore, protective mechanisms safeguard ventilation by offloading postural functions, thereby regulating pH and homeostatic balance (18). Although it was not possible to assess phrenic postural input, a diminished postural drive to the diaphragm, coupled with a modest ventilatory demand, would be a likely explanation for the lack of diaphragm fatigue noted after intense arm-crank exercise.

Although others have reported a relationship between the magnitude of reduction in P_ga,tw_ after intense whole-body exercise and the ventilatory output or work performed by the abdominal muscles (35), our data suggest that contractile fatigue of the major expiratory muscles is not entirely dependent on ventilation. No significant correlations were found between peak values for V˙O_2_ or V˙_E_ during constant-load exercise and the magnitude of abdominal muscle fatigue, in agreement with previous studies (40). Furthermore, force output of the abdominal muscles (∫P_ga_ · *f*_R_) during maximal arm-crank ergometry was substantially higher than values reported previously for maximal lower-limb cycle ergometry (756 vs ~600 cm H_2_O·s^−1^·min^−1^), despite a substantially lower minute ventilation during the former (99 vs 153 L·min^−1^) (35). It seems highly likely, therefore, that a substantial proportion of the abdominal muscle force output noted in the present study comprised additional, nonrespiratory work.

The abdominals do not exhibit the same respiratory modulation as the diaphragm (19) and, during dynamic upper-body exercise, might undergo excessive loading in carrying out a series of interrelated ventilatory and mechanical functions. As well as contracting to reduce EELV and expand tidal volume during exercise, the abdominals also contract isotonically to flex and rotate the vertebral column (10). Because severe exercise required a greater external power output than heavy exercise, the contribution of the abdominal muscles to locomotion was likely exacerbated. Indeed, neural drive to the rectus abdominis (EMG_ra_) and abdominal muscle pressure swings (ΔP_ga_) were significantly greater during severe versus heavy exercise. In an effort to quantify the respiratory muscle contribution to locomotor function, we compared the data from five respiratory cycles immediately before the cessation of exhaustive arm cranking (peak-exercise) to five respiratory cycles performed immediately after the abrupt cessation of exercise when ventilation was still relatively high. When the high thoracic loads imposed by severe-intensity arm cranking were relinquished, there was an abrupt increase in tidal volume (2.03 to 2.39 L), suggesting that arm cranking imposes a degree of constraint on the ribcage. Although still present after heavy exercise, the increase in *V*_T_ was more modest (1.75 to 1.88 L). More pertinent is that intrathoracic pressure swings and abdominal and diaphragm EMG activity were substantially reduced immediately after exercise cessation (see Fig. 3), suggesting that a substantial portion of the respiratory muscle activity during the arm-crank exercise was due to nonrespiratory loading of the thoracic complex (e.g., for posture and locomotion).

#### Consequences of abdominal muscle fatigue

Exercise-induced abdominal muscle fatigue did not appear to impede alveolar ventilation or systemic O_2_ content (P_ET_CO_2_ < 25 mm Hg and SpO_2_ > 98% over the final minute). However, end-expiratory (and end-inspiratory) lung volumes represented a significantly higher percentage of TLC from the first minute of severe exercise through to exhaustion (i.e., dynamic hyperinflation) (see Fig. 2). At these high operating lung volumes, the inspiratory muscles must overcome additional elastic loads presented by the lung and chest wall (1). Furthermore, the pressure-generating capacity of the inspiratory muscles is impaired at high lung volumes because of alterations in the length-tension characteristics of the diaphragm and the orientation and motion of the ribs (12). There is also an increased dependence on accessory inspiratory muscles at high lung volumes (44). Collectively, these changes in inspiratory muscle function would be expected to increase the O_2_ cost of breathing and elevate dyspnea (11).

Although our study was not specifically designed to address the underlying mechanisms of dynamic hyperinflation during upper-body exercise, our observations do merit brief discussion. First, the hyperinflation during severe exercise might have been attributable to the greater exercise ventilation at this intensity (relative to heavy exercise). However, others have observed elevated lung volumes during arm cranking performed by healthy subjects relative to leg cycling at similar ventilations (2). This suggests that the locomotor mechanics of upper-body exercise might directly influence operating lung volumes. It is unlikely that abdominal muscle fatigue *per se* caused the increase in EELV because the initial shift in lung volume occurred too early (<1 min) for contractile fatigue to be manifest (26). Moreover, prior fatigue of the abdominal muscles in healthy subjects was found to have no influence on operating lung volumes during subsequent high-intensity cycle exercise (36). It appears, therefore, that contractile fatigue of the abdominal muscles is not a causal factor in the control of operating lung volumes during exercise. As proposed previously (2), the abdominal muscle contribution to the regulation of EELV during upper-body exercise might be compromised by increased requirements to stabilize the torso.

## CONCLUSIONS

This study is the first to present objective evidence of abdominal muscle fatigue in response to severe-intensity upper-body exercise in normal, healthy men. However, there was no concurrent fatigue of the diaphragm after heavy or severe upper-body exercise. It seems likely that the ventilatory role of the diaphragm was prioritized during exercise and that upper-body exercise was of insufficient ventilatory stress to induce contractile fatigue of the inspiratory muscles. By contrast, the abdominal muscle fatigue after severe exercise was likely caused by a combination of mechanical, postural, and ventilatory demands induced by upper-body locomotor mechanics. This expiratory muscle multitasking might render the abdominal muscles particularly susceptible to contractile fatigue during intense upper-body exercise. Further research is needed to determine the implications of these findings for athletes involved in upper-body dominant sports and for clinical populations engaged in upper-body rehabilitation programmes.
